# Complete chloroplast genome sequence of *Carthamus tinctorius* L. from PacBio Sequel Platform

**DOI:** 10.1080/23802359.2019.1643799

**Published:** 2019-07-18

**Authors:** Zhi-Hua Wu, Rui Liao, Xiang Dong, Rui Qin, Hong Liu

**Affiliations:** Hubei Provincial Key Laboratory for Protection and Application of Special Plant Germplasm in Wuling Area of China, College of Life Science, South-Central University for Nationalities, Wuhan, China

**Keywords:** *Carthamus tinctorius*, chloroplast genome, PacBio Sequel, evolution

## Abstract

*Carthamus tinctorius* L, also known as safflower, is an important oil crop planted worldwide. The complete chloroplast (cp) genome was reported in this study using the PacBio Sequel Platform. The cp genome with a total size of 152,963 bp consisted of two inverted repeats (25,128 bp) separated by a large single-copy region (84,124 bp) and a small single-copy region (18,583 bp). Further annotation revealed the cp genome contains 112 genes, including 79 protein-coding genes, 29 tRNA genes, and 4 rRNA genes. The information of the cp genome will be useful for investigation of evolution and molecular breeding of safflower in the future.

Safflower (*Carthamus tinctorius* L.), an annual or biennial herb of Asteraceae, has been planted in China for more than 2000 years (Wu and Zheng 2004; Qin et al. 2019). The seeds of safflower are known for abundant linoleic acid which has protective effects from osteoporosis and rheumatoid arthritis, and their dried florets have been used for treating stroke and coronary heart disease in traditional Chinese medicine (Yu et al. 2013; Rapson et al. 2015). In this study, to get the new insight into the evolution of safflower, we sequenced, assembled, and annotated the accurate cp genome with PacBio Sequel platform.

The materials of safflower (Voucher specimens no. WH2018052810001, HSN, located at N 30°29′8″, E 114°24′3″) in this study were collected from Hubei Province, the voucher specimens were deposited at Herbarium of South-Central University for Nationalities (HSN). The total genomic DNA was extracted using a modified cetyltrimethylammonium bromide (CTAB) method and sequenced using the PacBio platform. The whole chloroplast (cp) genome was assembled using Canu-1.5 (Koren et al. 2017) and got 255 contigs with the N50 of 278,038 bp. To obtain the complete cp sequence, we aligned the contigs of a preliminary assembly to the whole cp data from NCBI. Then the draft genome was polished with Arrow (SMRT link-6.0.0, Pacific Biosciences, Menlo Park, CA). Due to the special structure of the cp genome, we mapped the scaffolds to the reference to find the IR region and manually adjusted. Then annotated using DOGMA (Wyman et al. 2004). The complete cp genome was 152,963 bp (MK983238) and composed of two inverted repeats (IRs) of 25,128 bp each, which divide a large single copy (LSC) region of 84,124 bp and a small single copy (SSC) region of 18,583 bp, the average GC content was 37.80%. The cp genomes encoded 112 functional genes, including 79 protein-coding genes, 29 tRNA genes, and 4 rRNA genes as well as 32 SSR markers.

According to the previously published cp genome of safflower from NCBI with Illumina platform (KX822074.1), we aligned the safflower cp of Illumina and PacBio platforms using BLASTN and found that the genome got from PacBio platform was slightly longer. After designing the primer (5′-AATGGGTCTGAGCGGAAAT-3′ and 5′-TCTAAAGACCGAGATGGA-3′) for different places between the genome with two platforms, we verified the real existence of the insertion assembled by PacBio through Sanger. The result showed that the PacBio has the advantage of getting more complete cp genome, which is also reported in other plants (Wu et al. 2014).

In our study, to explore the phylogenetic relationship of safflower within Asteraceae, additional 19 species from Asteraceae were studied. With two species of Cornales as the outgroup, the phylogenetic trees were built from the whole protein-coding gene matrix by maximum-likelihood (ML) and Bayesian inference (BI) (Figure 1). The ML tree was generated using IQ-TREE (Nguyen et al. 2015) based on the best model of GTR + F + R3 and 1000 bootstrap replicates, and BI analysis was performed in MrBayes-3.2.6. This result showed that the analyzed Asteraceae species were clustered into three clades and the safflower was located at Cardueae.

**Figure 1.**
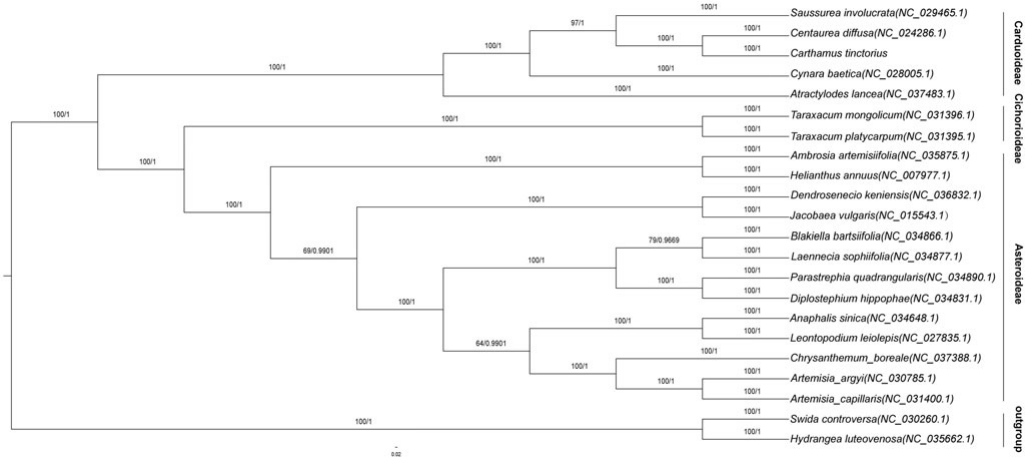
Phylogenetic tree inferred by both maximum-likelihood and Bayesian from 22 representative species, including safflower in our analysis, 19 of Asteraceae and two Cornales as outgroup from public data. The values on each node represent the bootstrap values from maximum-likelihood (left) and the posterior probability from Bayesian inference (right), respectively.
